# G-Quadruplex Formed by the Promoter Region of the *hTERT* Gene: Structure-Driven Effects on DNA Mismatch Repair Functions

**DOI:** 10.3390/biomedicines10081871

**Published:** 2022-08-03

**Authors:** Anzhela V. Pavlova, Victoria Yu. Savitskaya, Nina G. Dolinnaya, Mayya V. Monakhova, Anastasia V. Litvinova, Elena A. Kubareva, Maria I. Zvereva

**Affiliations:** 1 Department of Chemistry, Lomonosov Moscow State University, Leninskye Gory 1, 119991 Moscow, Russia; p.anzhela98@gmail.com (A.V.P.); svk1896@mail.ru (V.Y.S.); dolinnaya@hotmail.com (N.G.D.); 2 Belozersky Institute of Physico-Chemical Biology, Lomonosov Moscow State University, Leninskye Gory 1, 119991 Moscow, Russia; monakhovamv@gmail.com (M.V.M.); kubareva@belozersky.msu.ru (E.A.K.); 3 Department of Bioengineering and Bioinformatics, Lomonosov Moscow State University, Leninskye Gory 1, 119991 Moscow, Russia; nas-lit@bk.ru

**Keywords:** *hTERT* promoter, G-quadruplex, DNA mismatch repair, MutS, MutL

## Abstract

G-quadruplexes (G4s) are a unique class of noncanonical DNAs that play a key role in cellular processes and neoplastic transformation. Herein, we focused on the promoter region of human *TERT* oncogene, whose product is responsible for the immortality of cancer cells. It has been shown by chemical probing and spectroscopic methods that synthetic 96-nt DNAs modeling the wild-type G-rich strand of the *hTERT* promoter and its variants with G>A point substitutions corresponding to somatic driver mutations fold into three stacked parallel G4s with sites of local G4 destabilization caused by G>A substitutions in the G4 motif. These models were used to elucidate how the *hTERT* multiG4 affects the binding affinity and functional responses of two key proteins, MutS and MutL, involved in the initial stage of DNA mismatch repair (MMR) in *Escherichia* *coli* and *Neisseria* *gonorrhoeae* with different MMR mechanisms. We have shown for the first time that (i) point substitutions do not affect the effective binding of these proteins to the *hTERT* G4 structure, and (ii) the endonuclease activity of MutL from *N. gonorrhoeae* is significantly suppressed by the stable G4 scaffold. It is likely that some of the genomic instability associated with G4 may be related to the blockage of human intrinsic methyl-independent MMR attempting to operate near G4 structures.

## 1. Introduction

**Figure 1:**
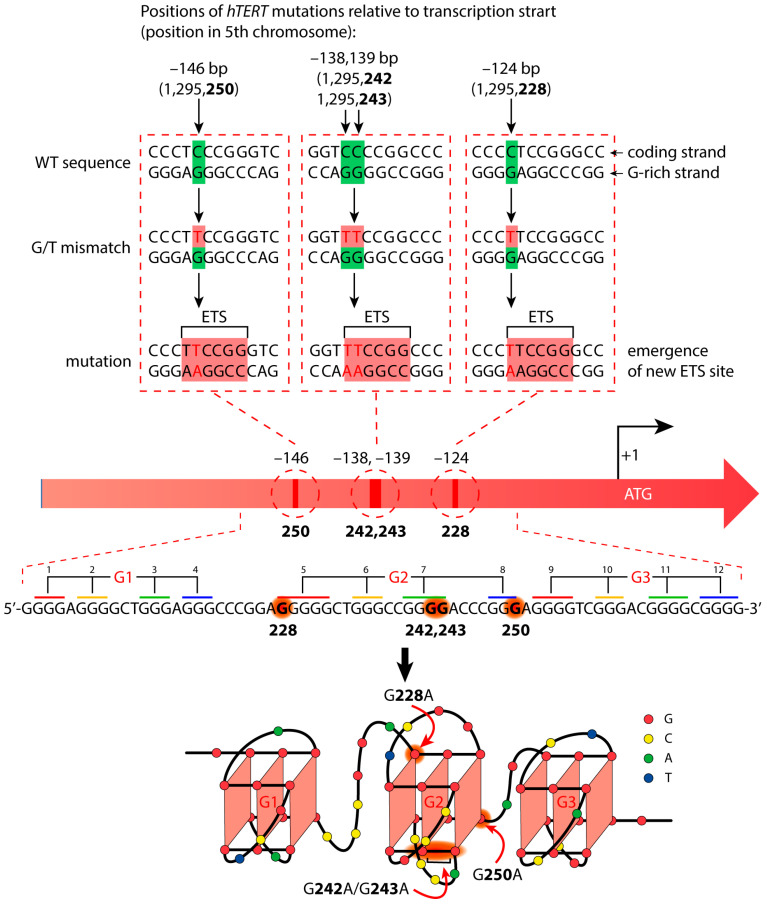
Cancer-specific mutations in the *hTERT* promoter’s primary structure that generate de novo binding sites for the ETS transcription factors and putative structure that was proposed for the G-rich (noncoding) strand of the *hTERT* promoter. Below is the sequence of the WT *hTERT* G4 motif and one possible way of G4 folding. The assignment of G-tracts (numbered) to three G4-motifs (G1, G2, and G3) is presented. Common mutation sites are indicated in red in the *hTERT* G-rich sequence and in red arrows in the structural model.

The extremely high mutation frequency in the noncoding regions of the genome is being actively studied to better understand the mechanisms of oncogenesis. One of the most studied is the promoter region of the human telomerase reverse transcriptase gene (*hTERT*). The hTERT enzyme is primarily responsible for the maintenance of telomeres, and its activity is thought to be vital for cell immortalization [1,2]. hTERT is normally inactive in somatic cells (apart from stem cells), and its aberrant expression is associated with 85–90% of the cancers investigated thus far [3,4]. The etiology of oncological processes is based on the accumulation of various mutations localized both in somatic cells and in germ lines. Functional genetic studies have identified several point mutations within the *hTERT* core promoter region (approximately −180 to +1 of transcription start site) (Figure 1) [5]. Mutually exclusive C>T substitutions in the coding *hTERT* promoter strand occur most frequently at positions −124 bp (position 1295228 of chromosome 5, the mutation is designated as C228T) and −146 bp (position 1295250—C250T) relative to the ATG start codon of the *hTERT* gene [6]. These mutations are found in 60–80% of urothelial carcinomas [7], 71% of melanomas [8], 83% of glioblastomas [9], and a variety of other cancers. A less common double substitution CC>TT at positions −138, −139 bp from the start codon (C242T, C243T) is found mainly in the genomes of skin tumor cells.

The C>T substitutions under consideration in the coding *hTERT* promoter strand generate G/T DNA mismatches that are effectively recognized and removed by the mismatch repair (MMR) system. The MMR system is known to be responsible for maintaining genetic information by correcting base substitutions and insertion-deletion mismatches generated during DNA replication [10]. Failure to repair the G/T mismatch will result in C/G>T/A mutations in the next round of DNA replication due to the replacement of a guanosine residue in the noncoding (template) G-rich strand of the *hTERT* promoter with adenosine (Figure 1). These mutations create additional binding sites, 5′-CCGGAA-3′/3′-TTCCGG-5′, for the E-twenty-six (ETS) transcription factor family (Figure 1) and confer a selective advantage to glioblastoma, liver, and bladder cancer cells through the allelic recruitment of the multimeric GA-binding protein (GABP) transcription factor specifically to the mutant *hTERT* promoter. The cancer-specific interaction of GABP with the *hTERT* core promoter mutations provides a mechanism utilized by many cancers to overcome replicative senescence [7,11]. Therefore, the MMR system deserves special attention when studying the *hTERT* mutant promoter.

The oncologically relevant G>A substitutions (namely, G250A, G228A, or G242A, G243A) are clustered in the 68-nt G-rich region of the *hTERT* promoter containing twelve G-tracts of three or more consecutive guanines that allow for the formation of G-quadruplex (G4) structures (Figure 1). G4s constitute one of the most widely studied alternative forms of DNA, whose presence throughout the eukaryotic genome has been rigorously proven [12,13,14]. In endogenous G4s, G-tracts fold intramolecularly to form stacked planar G-tetrads with guanine bases from each tract linked via Hoogsteen hydrogen bonds. The quadruplex core is additionally stabilized by the guanine O6 coordination of monovalent cations (usually K^+^). G4s can have a wide range of folds: some are highly dynamic, while others only take one conformation [15]. Under in vitro conditions, G4s adopt parallel, antiparallel, and hybrid (3 + 1) topologies characterized by different orientations of the four G-tracts. The secondary structure formed in vitro by the 68-nt G4-forming sequence (G4 motif) in the *hTERT* promoter has been a subject of controversy for a long time [16,17,18,19]. One model features a structure with three parallel G4s interacting with each other via stacking interactions of terminal G-tetrads, while the other represents two stacked individual quadruplexes, including parallel G4 and G4 adopting a mixed (3 + 1) topology with an unusual DNA hairpin formed by the 26-nt loop. According to a recent study, the major form corresponds to three stacked parallel G4 units (Figure 1) [6].

G4s, in cooperation with numerous cellular proteins and enzymes, are capable of fine-tuning key biological processes such as DNA replication, chromosome end protection, transcription, mutagenesis, DNA repair, and recombination [20,21,22]. The rationale for our study was the hypothesis that the high mutation frequency in the G-rich promoters of oncogenes may be associated with quadruplex-driven changes in the function of repair proteins. Indeed, G4s were shown to have diverse effects on the functioning of the major DNA repair systems: base excision repair (BER) and nucleotide excision repair (NER). While these noncanonical DNA forms stimulate the NER-mediated repair of UV-induced lesions such as pyrimidine dimers, they demonstrate opposite effects on the BER machinery by suppressing the removal of oxidative base lesions from G4 motifs [23,24]. Some of these data were obtained for oncogene promoters such as *VEGF* and *c-Myc*, which form only one intramolecular G4.

**Figure 2:**
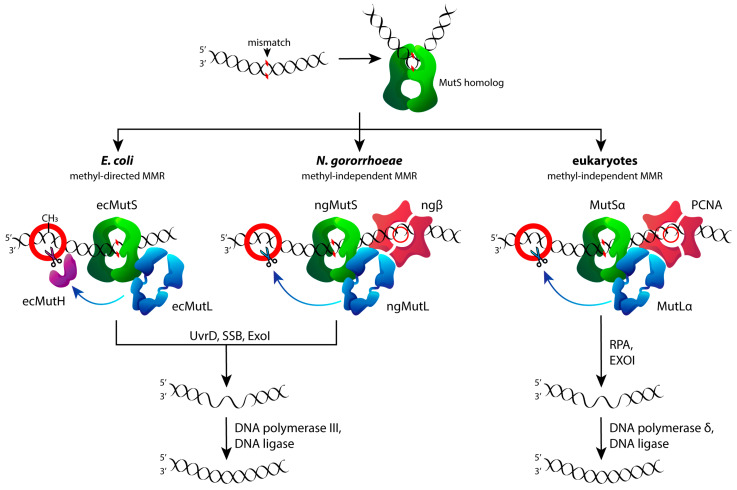
Mechanisms of the mismatch repair process in *E. coli* (**left**), *Neisseria gonorrhoeae* (**center**), and eukaryotes (**right**). In bacteria, homodimeric MutS recognizes mismatches and forms a complex at the site of the DNA mismatch. MutS then recruits MutL into the complex in an ATP-dependent manner. Similarly, in eukaryotes, heterodimeric MutSα is responsible for mismatch recognition and MutLα recruitment. In γ-proteobacteria (including *E. coli*), the MutS–MutL complex activates the MutH endonuclease, which nicks the unmethylated DNA strand (methyl-directed MMR). Most prokaryotes and all eukaryotes lack the *MutH* gene. In these organisms, MutL homologs (MutL homodimer for bacteria and MutLα heterodimer for eukaryotes) have endonuclease activity and selectively cut the newly synthesized strand (methyl-independent MMR). In this case, pre-existing nicks are important to distinguish the daughter strand.

At the same time, only a few facts of the interaction between the major MMR proteins (MutS and MutL homologs) and G4s have been established [25,26,27]. Generally, during the MMR process, the MutS protein initiates repair by scanning the DNA for a mismatch and recruits the MutL protein upon a mismatch detection. MutL then activates downstream repair, which involves a single-stranded break (nick) in the newly replicated strand, either by the MutH endonuclease in the methyl-directed MMR in *E. coli,* or by MutL itself in the methyl-independent MMR used by eukaryotes and most bacteria (Figure 2). *Escherichia coli* MutS (ecMutS), *Rhodobacter sphaeroides* MutS and human MutSα were reported to bind to various single G4 DNA with an affinity greater than that for the G/T mismatch, but such preferential binding does not correlate with DNA mismatch repair activity; moreover, the modes of MutS binding to a G4 and to a mismatched base pair appear to be different [23,25,26,27]. We recently showed that MutL from *E. coli* (ecMutL) binds to a single parallel G4 formed by the d(GGGT)_4_ sequence with a higher affinity than to double-stranded DNA. Despite the strong binding of MutS and MutL to G4, the latter is not recognized by *E. coli* MMR as a signal for repair, but the G4 does not prevent and even activates MMR processing when a G4 and G/T mismatch are in close proximity [23,27].

In this work, we focused on studying the impact of the specific higher-order *hTERT* G4 structure on the efficiency and accuracy of two MMR pathways. The *hTERT* G4 formation induces the recruitment G residues from G/T mismatches resulting from base substitution in the G/C-rich region of the promoter, which should directly affect MMR activity. A single-stranded 96-nt fragment of the *hTERT* promoter noncoding strand harboring twelve consecutive G-tracts was chosen as a model system. The effect of biologically and clinically significant nucleotide substitutions on the folding of G4 structures, as well as their recognition by MutS and MutL proteins, was studied using three constructed 96-nt DNA fragments with single (double) G>A substitutions (G250A, G228A, or G242A/G243A) (see above and Figure 1). For the first time, an attempt was made to find out whether the higher-order *hTERT* G4 structure could be hydrolyzed by the MutL with an endonuclease function, thereby contributing to genome instability. 

To gain this first insight into the role of higher-order G4 structures in the initial steps of MMR, we selected proteins from two bacterial sources, *E. coli* and *N. gonorrhoeae,* due to their availability and characteristics. The overall MMR mechanism is highly conserved across all organisms and proceeds through four main steps: lesion detection, the recognition of a mismatch-containing daughter strand, the degradation of the mismatch-containing DNA fragment, and DNA resynthesis and ligation (Figure 2). However, the MMR mechanisms of *E. coli*, on the one hand, and eukaryotes and most bacteria such as *N. gonorrhoeae,* on the other hand, have some differences. First, the signals for distinguishing the daughter and maternal DNA strands differ: the MutH-dependent recognition and nicking of the certain monomethylated site in DNA is characteristic for *E. coli*, while in eukaryotes and other prokaryotes, except for enterobacteria, MutL has an endonuclease function itself and discriminates DNA strands based on pre-existing nicks and gaps. Second, the eukaryotic homologs of the MutS and MutL proteins are heterodimers, while the MutS and MutL of *E. coli* and *N. gonorrhoeae* are homodimers. Despite these differences, MutS and MutL are highly conserved proteins, regardless of the strand discrimination mechanism. MutS homologs share a high affinity for DNA mismatches, the function of ATPase, which promotes DNA repair by stimulating conformational changes in the protein, and the ability to recognize and interact with noncanonical DNA, particularly G4 [28]. This fact is partly explained by the uniformity of the types of DNA damage in all organisms [29]. MutL homologs are more variable in evolution than MutS, although they still have a highly conserved ATPase N-terminal domain responsible for the interaction with DNA [30] (see Section 3.4 and Supplementary Figures S1–S3). In our study, * E. coli* MutS and MutL were used as a comparison standard associated with the early research data [27]. As for *N. gonorrhoeae,* this microorganism, in contrast to *E. coli*, uses the methyl-independent MMR mechanism [31] inherent in eukaryotic MMR systems. MutL from *N. gonorrhoeae* (ngMutL), like the human MutL protein, has endonuclease activity and introduces a nick in double-stranded DNA (Figure 2).

It has been shown by chemical probing and spectroscopic methods that the 96-nt DNAs modeling the G-rich strand of the human *TERT* promoter and its variants with G>A point substitutions corresponding to somatic driver mutations fold into three stacked parallel G-quadruplexes with a local destabilization in the site of the substitution. MutS and MutL from *E. coli* effectively bind to the *hTERT* quadruplex structure despite the presence of point nucleotide substitutions. Tandem *hTERT* G4s are recognized and strongly bound by MutL from *N. gonorrhoeae*, but significantly suppress its endonuclease activity.

## 2. Materials and Methods

### 2.1. DNA Oligonucleotides

All oligodeoxyribonucleotides (synthesized via standard phosphoramidite chemistry and purified by high-pressure liquid chromatography in Syntol, Russia) were used without further purification. Oligonucleotide strand concentrations were determined spectrophotometrically by means of extinction coefficients derived from the nearest-neighbor data [32].

### 2.2. Preparation of Intramolecular G4 Structures and DNA Duplexes

WT and G>A-substituted *hTERT* G4s were prepared by annealing the 96-nt DNA samples in harsh conditions, including those labeled with tetramethylrhodamine (TAMRA), via heating them at 95 °C for 20 min and cooling them overnight to 4 °C in appropriate buffers containing at least 20 mM KCl. Control G4 structures carrying one quadruplex unit were generated using a standard annealing procedure.

To prepare DNA duplexes, complementary DNA strands were annealed (by heating at 95 °C for 5 min and slowly cooling to room temperature) in an appropriate buffer solution; the unlabeled strand was used in a 5% excess compared to the TAMRA-labeled strand.

### 2.3. Purification of Recombinant Proteins

Proteins ecMutS, ecMutL, and ngMutL were purified as described previously [33,34]. The recombinant proteins were expressed as *N*-terminal His_6_-tagged proteins in *E. coli* strain BL21(DE3) and purified by Ni-NTA affinity chromatography. Purification was followed by size exclusion chromatography on a Superdex 200 TM 10/300 (GE Healthcare, Chicago, IL, USA) on an Äkta Purifier (GE Healthcare, Chicago, IL, USA). The resulting proteins were aliquoted, frozen in liquid nitrogen, and stored in 10 mM HEPES-KOH buffer (pH 7.9) with 200 or 300 mM KCl, 1 mM EDTA, 10% (*v*/*v*) glycerol, and optionally, 1 mM 2-mercaptoethanol at −80 °C.

The β-subunit of DNA polymerase III (ngβ) was purified similarly to ecMutS, ecMutL, and ngMutL by Ni-NTA affinity chromatography. The ngβ sample was further purified on HiTrap Heparin HP (1 mL) column pre-equilibrated in 10 mM K-phosphate buffer (pH 7.5) with 0.5 mM EDTA. The protein was eluted with a salt gradient to 0–0.5 M KCl as previously described [35]. The ngβ fractions were dialyzed in a 10 mM HEPES-KOH buffer (pH 7.9) with 200 mM KCl, 1 mM EDTA, and 10% (*v*/*v*) glycerol; then, they were aliquoted, frozen in liquid nitrogen, and stored at −80 °C.

Protein concentration in the fractions was determined spectrophotometrically with a NanoDrop ND-1000 (Thermo Fisher Scientific, Waltham, MA, USA) at 280 nm. Extinction coefficients were calculated with ProtParam Tool [36].

### 2.4. A Chemical Probing Assay

Chemical modification of 3′- and 5′-TAMRA–labeled single- or double-stranded DNA samples (Supplementary Table S1) with dimethyl sulfate (DMS) was performed according to the standard procedure. Namely, 20 pmol of single- or double-stranded DNA annealed in the presence of 100 mM KCl was incubated for 2 min at 25 °C with 7 nmol of DMS in 100 μL of 50 mM sodium cacodylate buffer (pH 8.0) containing 100 mM KCl and 0.5 mg/mL tRNA. The reaction was stopped by adding 20 μL of 10% 2-mercaptoethanol in 100 mM NaOAc solution. The modified DNA was precipitated with an ethanol–NaOAc solution and cleaved with 10% piperidine at 90 °C for 30 min. After evaporation of the piperidine, the cleavage products were dissolved in 70% formamide and separated by electrophoresis in a 20% polyacrylamide gel containing 7 M urea. DNA fragments were visualized with Typhoon FLA 9500 (GE Healthcare, Chicago, IL, USA).

### 2.5. UV Melting Experiments

Unlabeled single-stranded 96-nt DNA sample containing the WT *hTERT* G4 motif with single-stranded flanks (Supplementary Table S1) was annealed in 8 mM K-phosphate buffer (pH 7.1) containing 20 mM KCl. Absorbance-versus-temperature profile of DNA oligonucleotide (at ~4 µM concentration per oligonucleotide strand) were recorded in a 600 μL quartz microcuvette (Hellma Analytics, Müllheim, Germany) with an optical path length of 10 mm on a double-beam Hitachi U-2900 UV/visible spectrophotometer (Hitachi, Tokyo, Japan) equipped with a Hitachi thermoelectric controller. Changes in absorbance were monitored between 20 and 85 °C at 295 nm at a heating rate of 0.5 °C/min. Melting temperature (T_m_), defined as the temperature of the mid-point, was estimated from a minimum value of the first derivative of the fitted curve for data smoothed with the Savitzky–Golay filter.

### 2.6. Circular Dichroism Measurements

CD spectra of unlabeled single-stranded WT *hTERT* G4-containing 96-nt DNA sample (Supplementary Table S1) annealed in 8 mM K-phosphate buffer (pH 7.1) containing 20 mM KCl were recorded in a quartz cuvette of 10 mm optical path length between 30 and 85 °C in temperature intervals of ~5 °C at the average heating rate of 0.5 °C/min on a Chirascan CD spectrometer (Applied Photophysics Ltd., Leatherhead, UK) equipped with a Peltier controller. The DNA concentration was chosen to attain absorption of 0.5–0.6 at 260 nm, which yields an optimum signal-to-noise ratio. The measurements were performed in the 220–360 nm wavelength range at a scanning speed of 30 nm/min and a signal averaging time of 2 s with constant flow of dry nitrogen. All the CD spectra were baseline-corrected for signal contributions caused by the buffer. CD spectra were plotted as a molar ellipticity per oligonucleotide strand against wavelength. The spectra were processed with the Origin 8.0 software (Electronic Arts, Redwood City, CA, USA) using the Savitzky–Golay filter. The CD melting profiles revealed the temperature dependence of the CD signal at 260 nm.

### 2.7. DNA-Binding Activity of ecMutS

Single-stranded 5′-TAMRA-labeled DNA probes (5 nM) (Supplementary Table S1) were incubated at 37 °C for 15 min with 2.5–160 nM ecMutS (per dimer) in 20 mM HEPES–KOH buffer (pH 7.9) containing 5 mM MgCl_2_, 120 mM KCl, 0.5 mg/mL BSA, and 1 mM nucleotide cofactor (ADP). Samples (10 μL) were analyzed in a 6% polyacrylamide gel under nondenaturing conditions in TAE buffer at 4 °C. Relative intensity of the DNA bands on electropherograms obtained with Typhoon FLA 9500 (GE Healthcare, Chicago, IL, USA) was evaluated using the TotalLab TL120 software (Nonlinear Dynamics Ltd., New Castle, UK). The percentage of DNA in a DNA–protein complex was determined as the ratio of the fluorescence intensity corresponding to the band of the ecMutS–DNA complex to the total fluorescence intensity of labeled DNA. All experiments were performed in triplicate. Apparent dissociation constants (*K*_D_^app^) corresponding to the ecMutS concentration at which 50% of the DNA ligand was complexed with the protein were averaged. Error bars represent the 95% confidence interval.

### 2.8. DNA-Binding Activity of ecMutL and ngMutL

Single-stranded TAMRA-labeled DNA probes (20 nM) (Supplementary Table S1) were incubated for 10 min on ice with 20–700 nM ecMutL or 15–500 nM ngMutL (per dimer) in 20 mM HEPES buffer (pH 8.0) containing 100 mM KCl, 0.5 mg/mL BSA, and 1 mM DTT. Samples (10–20 μL) were analyzed by electrophoresis in a 4 or 6% polyacrylamide gel in TAE buffer for 2–3 h at 4 °C. The efficiency of protein–DNA complex formation was evaluated in the same way as in assessing of the DNA-binding activity of ecMutS. Error bars represent the 95% confidence interval.

### 2.9. Hydrolysis of DNA by ngMutL

The ngMutL endonuclease activity was measured as described previously [37]. Reaction mixtures (10 μL) contained 20 mM HEPES-KOH buffer (pH 8.0), 100 mM KCl, 5 mM MgCl_2_, 5 mM MnCl_2_, 0.8 mM ATP, 0.5 mg/mL BSA, 12% (*v*/*v*) glycerol, 10 nM 3′-TAMRA-labeled single- or double-stranded DNA (Supplementary Table S1), and 250 nM ngMutL or 350 nM ngMutL (per dimer) in the absence and presence of equimolar amounts of the ngβ dimer. Samples were incubated at 37 °C for 90 min and quenched by the addition of 50 mM EDTA and 1 mg/mL proteinase K followed by incubation at 55 °C for 20 min. The products were electrophoresed in a 12% denaturing polyacrylamide gel containing 7 M urea. Gels were visualized using a Typhoon FLA 9500 (GE Healthcare, Chicago, IL, USA). The analysis of hydrolysis products was carried out using the TotalLab TL120 software. Error bars represent the 95% confidence interval.

### 2.10. Bioinformatic Analysis of MutS and MutL Sequence and Structure

The sequences of MutS homologs (ecMutS, ngMutS, MSH2, and MSH6) and MutL homologs (ecMutL, ngMutL, MLH1, and PMS2) were downloaded from the UniProt database according to their IDs. The protein sequences were aligned with the JalView program using ClustalO algorithm with default settings. The consensus motifs for MutS and MutL homologs were identified according to previous works [38,39]. The percentage of identical and similar amino acid residues for MutS and MutL homologs were obtained using an online resource [40] and Smith–Waterman algorithm with default settings.

Superposition of the 3D structures of ecMutS, MSH2, and MSH6 (PDB IDs 1E3M and 2OF8) was obtained in PyMOL (version 2.4.1, 2020-09-14, Schrödinger Inc., New York, NY, USA) using the /align command. Superpositions for N-terminal domains of ecMutL, MLH1, and PMS2 (PDB IDs 1BKN, 3NA3, and 1H7U) and C-terminal domains of ecMutL, ngMutL, and MLH1 (PDB IDs 1X9Z, 3NCV, and 3RBN) were obtained in the same program using the/super command.

## 3. Results

### 3.1. Design of the DNA Models

**Figure 3:**
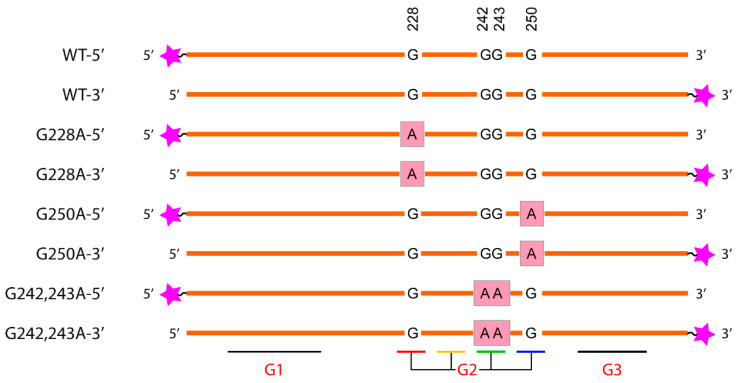
Schematic representation of a single-stranded 96-nt DNA fragment of the WT *hTERT* promoter (designated as WT-5′ or WT-3′ depending on the position of the TAMRA fluorophore) and its altered versions containing the G228A substitution (G228A-5′ or G228A-3′), the G250A mutation (G250A-5′ or G250A-3′), and double substitutions G242A/G243A (G242,243A-5′ or G242,243A-3′). Pink asterisks denote TAMRA attached to the 5′- or 3′-ends of the designed oligonucleotides. The schematic arrangement of G>A substitutions within the G-tracts is mapped below.

To monitor the G4 *hTERT*-mediated responses of the MMR proteins, we designed a 96-nt *hTERT* promoter fragment with a conserved 68-nt G4 motif harboring 12 G-tracts flanked by 14-nt sequences, as well as its altered versions containing the most common G>A substitutions at the G4-forming sequence (Figure 3).

It should be noted that the G>A substitutions in the *hTERT* promoter fall into the G-tracts 5, 7, and 8, corresponding to the second G4 motif, which ensures the formation of the central putative G4 unit in the general *hTERT* G4 conformation, which is the main subject of discussion.

For comparison, single-stranded 95-nt DNA with a d(GGGT)_4_ insert that facilitated the formation of a single G4 in the middle of the molecule (95G4) was used. The DNA duplex models: control ds96/96*, duplexes containing the *hTERT* G4 motifs (WT-3′/WT-C or WT-5′/WT-C), the corresponding duplexes with G>A substitutions in the *hTERT* promoter region, as well as 76 bp DNA duplexes that do not contain G4 motifs, were prepared by the hybridization of two fully complementary strands (the primary structures of the single- or double-stranded (ss or ds) oligonucleotide models used in this study are shown in Supplementary Table S1).

### 3.2. Thermal Stability and Topology of the G4 Structure Formed by the WT hTERT Promoter Region

**Figure 4:**
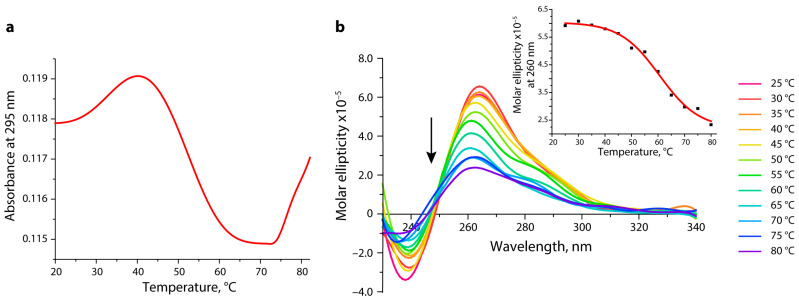
Estimation of the thermal stability and topology of the G4 structure folded by 96-nt WT *hTERT* promoter fragment by spectroscopic methods. (**a**) Temperature-dependent changes in UV absorbance at 295 nm monitored from 20 °C to 85 °C. (**b**) CD spectra recorded at different temperatures rising along the direction of the arrow; temperature values varied from 25 °C to 80 °C with an interval of 5 °C. (Insert) CD-monitored melting profile for WT *hTERT* G4 at 260 nm. All measurements were performed in an 8 mM K-phosphate buffer (pH 7.1) containing 20 mM KCl.

The characteristic features of the temperature dependence of UV absorption at 295 nm (the hypochromic effect and a sigmoidal shape of melting profile) [41] indicate that the G4 structure formed by the unlabeled 96-nt WT *hTERT* promoter (WT, Supplementary Table S1) unfolds with a T_m_ of 54 °C in a buffer solution containing 20 mM KCl (Figure 4a) after a prolonged annealing procedure (see Section 2). The increase in the UV absorption in the regions of low-temperature and high-temperature plateaus is explained by the disruption of the single-stranded stacking interactions in the unstructured 14-nt DNA fragments flanking the G4 motif. In the same sample, no G4 melting was observed in the temperature range from 20 °C to 85 °C when the KCl concentration was reduced to 5 mM (data not shown). The melting curves of the WT *hTERT* G4 showed a single-step cooperative transition. It can be assumed that the observed melting curve is a superposition of the melting of three quadruplex units, given that the major form of the multiquadruplex WT *hTERT* G4 structure corresponds to three stacked parallel G4 units (Figure 1) [6] that are stabilized by the same number of G-tetrads. In the case of a two-quadruplex model [16], the stability of each G4 should vary due to the different conformation and topology of the constituent components.

To confirm the existence of the G4 structure folded within the WT *hTERT* G4 and to determine its topology, CD measurements were performed under the same experimental conditions. The CD spectra displayed characteristics of a parallel G4 topology, manifesting in a positive band at ~265 nm followed by a negative band at 240 nm (Figure 4b). These spectral features clearly indicate the absence of an antiparallel G4 topology in the WT *hTERT* G4, independently confirming its three-quadruplex folding mode. Similar to the UV melting profile, the S-shaped dependence of the CD-mediated WT *hTERT* G4 melting curve observed at 260 nm showed a single-step transition with T_m_ ~59 °C (Figure 4b, insert). The T_m_ values found by both methods are quite close to each other, thereby confirming an independent evaluation of the WT *hTERT* G4 structure.

### 3.3. Chemical Probing Assays of WT and Altered hTERT G4 Structures

A comparative analysis of the WT and the altered *hTERT* G4 structures with point nucleotide substitutions was performed by DMS-induced chemical probing. This reagent methylates the N7 of guanines most effectively, resulting in a facile depurination and strand cleavage after a subsequent treatment with piperidine. This nitrogen is occluded in G-tetrads; therefore, DMS protection is widely utilized to evaluate the formation of G4 [42]. Potentially, G>A substitutions in the G4 motif can prevent the formation of G4 or change its structure and stability. A single-stranded 96-nt DNA fragment of the WT *hTERT* promoter and its three altered versions containing cancer-associated single or double G>A substitutions in G-tracts 5, 7, and 8 of the G2 motif are schematically presented in Figure 1 and Figure 3 along with their designations. The model DNAs differ not only in the site of the G>A substitution, but also in the position (5′- or 3′-terminal) of the TAMRA fluorophore. TAMRA-labeled oligonucleotides were used to visualize the DNA cleavage products after their separation in a polyacrylamide gel (denaturing conditions). The primary structures of the WT and altered versions of the 96-nt *hTERT* promoter fragments, as well as their hybridization products with fully complementary strands (DNA duplex models), can be found in Supplementary Table S1. Chemical probing experiments were performed in the presence of 100 mM potassium ions to facilitate the G4 formation.

**Figure 5:**
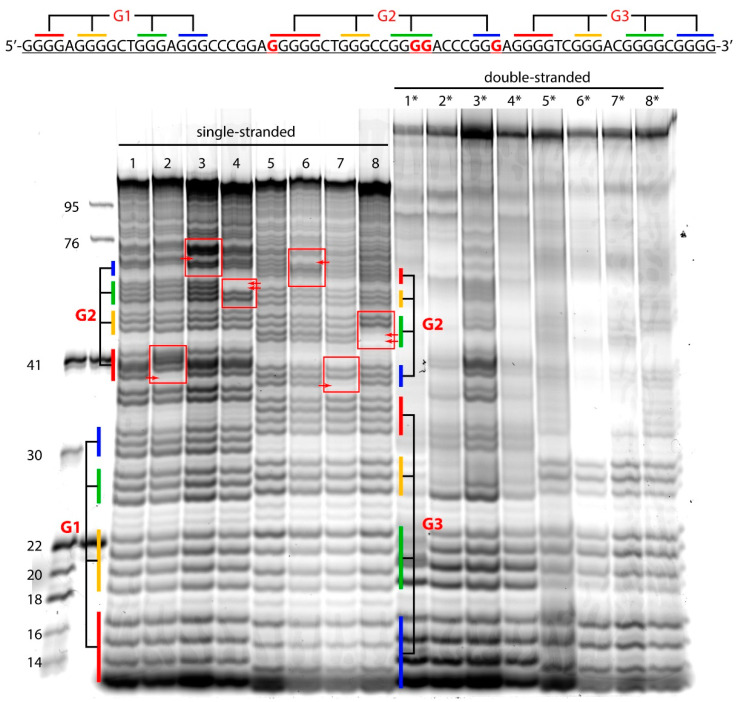
DMS-induced chemical cleavage patterns of single-stranded (lanes 1–8) and double-stranded (lanes 1*–8*) DNA models labeled with the TAMRA fluorophore at the 3′ ends (lanes 1–4 and 1*–4*) and 5′ ends (5–8 and 5*–8*). Lanes: 1—WT-5′, 2—G228A-5′, 3—G250A-5′, 4—G242,243A-5′, 5—WT-3′, 6—G228A-3′, 7—G250A-3′, and 8—G242,243A-3′. Lanes 1*–8* correspond to double-stranded models formed by 96-nt DNAs shown in the left panel and fully complementary oligonucleotides. The *hTERT* promoter sequences corresponding to the G-tracts forming each of the three G4 units (G1, G2, and G3) are color-coded in the electropherogram and in the primary structure shown above. Sites of substituted G residues are highlighted by red frames; low-intensity bands corresponding to G>A substitutions are indicated by red arrows.

According to our CD and UV-spectroscopy data, the WT *hTERT* promoter fragment does not form the two-quadruplex structure with the hybrid (3 + 1) topology postulated for one of the G4 units, but rather folds into a compact stacked three-G4 conformation. Since the used G>A substitutions in the *hTERT* promoter are clustered in the central quadruplex within this structure, we focused on a comparative analysis of the degree of DMS protection in this G4 unit, referred to as G2 in Figure 5.

The DMS probing results (Figure 5) suggest that the formation of a stacked three-G4 structure actually occurs in the ss WT and altered 96-nt DNA fragments of the *hTERT* promoter. The guanine residues from the G1 and G3 quadruplex-forming sequences have a clearly lower reactivity toward DMS compared to the same guanines in the double-stranded ones (compare the left and right panels in Figure 5). The poor resolution of the long cleavage products of the DNA duplexes, even in the harsh denaturing conditions of the gel electrophoresis, did not enable a robust analysis for the G-tracts of the G2 unit. However, as expected, hyperreactive guanine bases can be seen at the border between the G4 motif and the flanking DNA sequence, since the central guanines are more protected from the reaction with DMS than the outer ones. These data provide good evidence for the involvement of guanines from the G4 motifs in the 96-nt *hTERT* promoter involved in Hoogsteen base pairing.

The comparison of the DMS cleavage intensity of the WT *hTERT* promoter region and its altered versions showed a significantly higher reactivity of guanine residues located near the G>A substitutions within the G2 motif (compare lanes 1 and 2–4, 5, and 6–8 in Figure 5), which indicates their increased exposure to the reagent due to a local destabilization of the G4 structure. At the same time, an attenuated chemical cleavage was observed for the G>A substitutions themselves (indicated by red arrows in Figure 5), since adenine residues are less reactive towards DMS than guanines; these data confirm the presence of G>A substitutions in the studied DNA samples.

It should be noted that the G228A and G250A as well as the G242A/G243A substitutions are located in G-tracts containing five, three, and four consecutive dG residues. As evident from the results, the guanines flanking the G250A substitution in the shortest three-nucleotide G-tract showed significantly greater cleavage as they were more exposed to the DMS methylation due to local G4 destabilization (compare lanes 3 and 1 in Figure 5). In addition, the G250A substitution was found to contribute to the high reactivity of the guanine residue belonging to the 9th G-tract from the neighboring G3 motif (Figure 1), i.e., this substitution can cause a rearrangement of part of the *hTERT* multimeric G4 structure by recruiting dG from the adjacent quadruplex-forming sequence.

### 3.4. The Structural Similarity of Prokaryotic MutS and MutL with Eukaryotic Homologs

To study the effect of the *hTERT* promoter G4 on DNA mismatch repair function, two key MMR proteins, MutS and MutL, were chosen. MutS acts as a DNA sensor to detect mismatches, including G/T, C/T, A/C, A/G, G/G, A/A, and T/T (all but C/C), as well as small insertion/deletion loops [43]. The most studied is ecMutS. Its quaternary structure in solution is an equilibrium mixture of dimers and tetramers formed by the equivalent subunits [44]. In eukaryotes, the heterodimers known as MutSα (MSH2–MSH6) and MutSβ (MSH2–MSH3) together function as the bacterial MutS protein, ensuring the fidelity of mitotic replication [45].

A central role in coordinating the various stages of MMR is assigned to the MutL protein. MutL receives a mismatch detection signal and directs excision repair in the daughter DNA strand and DNA repair synthesis [46]. MutL, similar to MutS, functions as a dimer. The heterodimeric MutLα (MLH1-PMS2) is a functional homolog of the ecMutL homodimer in eukaryotes.

Biochemical studies of the MutSα and MutLα proteins are complicated. The latter is attributed to its heterodimeric structure, which makes it difficult to obtain various subunits in an equimolar ratio [47]. To isolate pure MutSα and MutLα, a multi-step purification is required, as other DNA-binding proteins can be co-isolated along with the subunits of MMR proteins [48]. We argue that their bacterial homologs can be used for a preliminary analysis of the interaction between *hTERT* G4 and human MutSα and MutLα. Our experiments were performed with MutS and MutL from *E. coli* and MutL with endonuclease function from *N. gonorrhoeae*.

**Figure 6:**
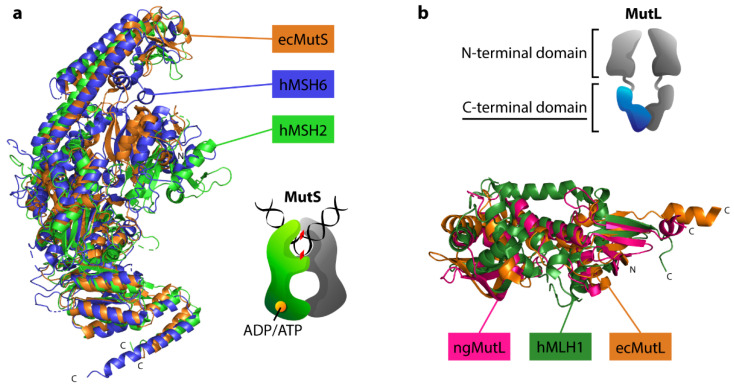
Superposition of crystal structures of MutS and MutL homologs. (**a**) Superposition of ecMutS (PDB ID 1E3M) and MSH6/MSH2 MutSα subunits from *H. sapiens* (PDB ID 2OF8). ecMutS, hMSH2, and hMSH6 are colored orange, light-green, and blue, respectively. (**b**) Superposition of hMLH1, ngMutL, and ecMutL C-terminal domains (PDB IDs 3RBN, 3NCV, 1X9Z, respectively). hMLH1, ngMutL, and ecMutL are shown in green, pink, and orange, respectively.

We aligned the amino acid sequences of ecMutS, MSH6, and the MSH2 of *H. sapiens* (hMSH6 and hMSH2) to compare their conserved motifs and residues (Supplementary Figure S1). According to our alignment, ecMutS and hMSH6 share around 28% of identical residues. The ATPase domain present in all MutS homologs is the most conserved with respect to the ITGPNMGGKSTY(L)M(I)RQ motif, which is inherent in all three analyzed sequences. A characteristic motif, GD(K)FYE(T), located in the N-terminal mismatch recognition domain was also found for ecMutS, hMSH6, and hMSH2. Overlaying the 3D structures of these proteins shows that they match well. The most similar are the tertiary structures of ATPase, the core domains, and the helix-turn-helix motif (Figure 6a).

The MutL sequence was found to contain 16 conserved motifs [39]. We aligned the ecMutL and ngMutL sequences with the human MLH1 (hMLH1) and PMS2 (hPMS2) protein sequences. The alignment of the four analyzed MutL sequences revealed the existence of a group of very conserved motifs: II, III, IV, V, VII, and X (Supplementary Figure S2). These motifs are located in the *N*-terminal domain of MutL and are involved in ATP binding and hydrolysis. It should be noted that ngMutL and hPMS2 have an endonuclease function, while ecMutL and hMLH1 do not. Conservative motifs in the C-terminal domain are responsible for DNA nicking. Pairwise alignments of MLH1 and ecMutL sequences, as well as the hPMS2 and ngMutL sequences, showed approximately 28% and 22% identity, respectively.

A superposition of the available crystal structures of *N*-terminal domains of ecMutL and MLH1 showed a good match with each other (Supplementary Figure S3). Interestingly, a comparison of the structures of the ngMutL C-domain with the C-domains of hMLH1 and ecMutL lacking the endonuclease function also showed a high similarity of their tertiary structures (Figure 6b).

### 3.5. Interaction of ecMutS and ecMutL with the WT hTERT G4 and Its G>A Substituted Analogs

**Figure 7:**
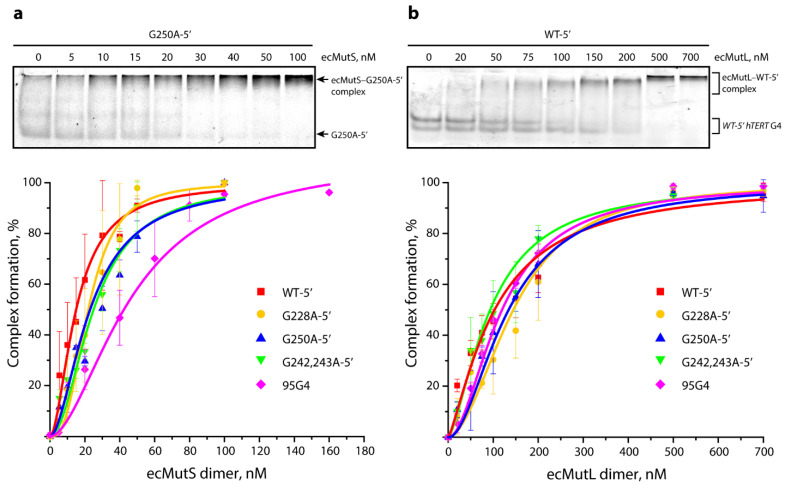
Binding of ecMutS (**a**) and ecMutL (**b**) to ssDNA models containing G4 motifs. Representative electropherograms of ecMutS and MutL binding to *hTERT* G4s, including G250A-5′ and WT-5′, respectively, are shown above. Below, the yield of nucleic acid–protein complexes, as calculated from the data of the EMSA, is plotted against the total protein concentration: 0–160 nM per ecMutS dimer or 0–700 nM per ecMutL dimer at 5 nM DNA or 20 nM DNA, respectively.

MutS plays a central role in mismatch detection but also interacts with some non-B form structures of DNA. In our previous study, we demonstrated that ecMutS, depending on the nucleotide cofactor, binds to a single parallel G4 structure with the same or even higher efficiency compared to mismatched DNA [27]. Here, we analyzed the binding affinity of ecMutS (methyl-directed mechanism) to a single-stranded 96-nt WT *hTERT* G4 and its G>A-substituted analogs and a 95-nt 95G4 model containing a single parallel G4 structure in the middle of molecule formed by the d(GGGT)_4_ insert. We studied the DNA•ecMutS complex formation in the presence of ADP, monitored by an electrophoretic mobility shift assay (EMSA). The DNAs were labeled with the TAMRA fluorophore at the 5′ ends. The curves of ecMutS’s direct binding to the DNA ligands are presented in Figure 7 and Figure S4, and the corresponding values of the apparent dissociation constants (*K*_D_^app^) values are presented in Table 1. Additional competitive binding experiments for the ecMutS and *hTERT* G4 variants confirmed the direct binding results (see Supplementary Figure S5 and Table S2 for details).

Under the conditions used (see Section 2), no significant difference was observed in ecMutS’s binding to the WT and the altered *hTERT* G4s, thus confirming the formation of very similar G4 structures, regardless of the presence of G>A substitutions in the central G4 motif and their location. Interestingly, the binding efficiency of ecMutS to DNA with a single G4 formed by the d(GGGT)_4_ insert was 2.5 times lower than *hTERT* WT-5′, containing three tandem stacked G4 units (Table 1, Figure S6).

We then investigated the direct binding of the entire set of TAMRA-labeled, ss96-nt *hTERT* G4 models to the ecMutL protein using the EMSA assay. The data obtained are shown in Figure 7b as the dependence of ecMutL’s binding efficiency on total protein concentration; the corresponding *K*_D_^app^ values are presented in Table 1. Compared to ecMutS, ecMutL, which serves as a molecular matchmaker, has weaker DNA-binding activity, which is largely dependent on the DNA length [49]. It is likely that the almost identical length of the DNA substrates (96 and 95 nt) was the reason why the binding affinity of ecMutL to the WT-5′ *hTERT* G4 and 95G4 was almost the same, although the latter contains a single G4 motif, d(GGGT)_4_, instead of the three motifs in the WT-5′ *hTERT* G4. At the same time, no significant difference was observed in the binding of both ecMutS and ecMutL to the WT and the altered *hTERT* G4s, suggesting these MMR proteins recognize the G4 conformation through a structure-based mechanism.

### 3.6. Binding Affinity of ngMutL to the WT hTERT G4 and Its Altered Variants; ngMutL-Mediated Processing of DNA Substrates Containing Multiquadruplex Structures

Compared to ecMutL, the ngMutL protein from the *N. gonorrhoeae* repair system, which uses a methyl-independent mechanism inherent in most bacterial and human MMR, may have additional responses to G4 DNA structures [50]. In contrast to *E. coli*, in organisms lacking MutH (e.g., *N. gonorrhoeae*, *R. sphaeroides*, human, etc.), the C-terminal domain of MutL exhibits a weak endonuclease activity, nicking the newly synthesized DNA strand during mismatch correction [34]. Herein, we examined the binding affinity of ngMutL to WT and altered *hTERT* G4s and evaluated the efficiency of ngMutL-mediated cleavage of the *hTERT* G4-containing DNA substrates.

The effect of the G4s in the ss96-nt *hTERT* promoter fragments on the recognition and binding to the ngMutL protein was assessed by an EMSA assay using 3′-TAMRA-labeled DNA models. For comparison, a ss 95-nt DNA with one parallel G4 in the middle of the molecule (95G4) and a 96-bp DNA duplex without G4 motifs (ds96/96*) were used. The primary structures of all the studied oligonucleotide models are shown in Supplementary Table S1. The data obtained are shown in Supplementary Figure S7 as a dependence of the DNA binding efficiency on ngMutL concentration; the corresponding *K*_D_^app^ values are presented in Table 2. As shown earlier, the binding efficiency of ngMutL for ssDNA is somewhat reduced (by 1.5 time) compared to the double-stranded ones [51].

According to the data obtained, the ngMutL protein binds more efficiently to DNA fragments containing G4 compared to ds96/96* lacking this noncanonical structure. However, the number of quadruplex units in DNA does not significantly affect the dissociation constants (compare the data for 95G4 and WT-3′ *hTERT* G4). The *K*_D_^app^ values range from 41 to 51 nM for the WT-3′ *hTERT* G4 and its altered forms, indicating the formation of stable protein-nucleic acid complexes. Interestingly, a significant difference was not observed for the ngMutL binding to the WT and G>A-substituted *hTERT* G4s, which confirms the formation of closely related G4 structures, regardless of the presence of a G>A single or double nucleotide substitution in the G4 central motif and its location. The same features were observed for the ecMutS and ecMutL proteins.

It was previously shown that the binding motif of the β-subunit of DNA polymerase III (β-clamp), encoded in the endonuclease domain of ngMutL, promotes β-clamp-dependent nicking activity towards the nascent DNA strand in a linear substrate [52]. We tested the endonuclease activity of ngMutL in the presence of equimolar amounts of β-clamp from *N. gonorrhoeae* (ngβ), as described earlier [37]. At the first stage, the 3′-TAMRA-labeled ss76-nt oligonucleotide of a random sequence and the 76-bp DNA duplexes with and without a G/T mismatch, referred to as ds76G/T and ds76A/T, respectively (Supplementary Table S1), were used to choose the optimal cleavage conditions. The ngMutL-mediated DNA cleavage (for ssDNA) or nicking (for dsDNAs) resulted in shorter labeled oligonucleotide cleavage products, which were separated from the intact DNA substrate by gel electrophoresis in denaturing conditions, enabling an evaluation of the cleavage efficiency (Supplementary Figure S8). We have shown that under the selected conditions (See “Section 2”), the presence of a G/T mismatch does not significantly affect the ngMutL-mediated nicking of the 76-bp DNA duplexes (compare lines 4 and 6 in Supplementary Figure S8). This is consistent with the generally accepted understanding of the DNA mismatch repair mechanism, where the MutS is a key sensor for mismatch recognition [43]. It is important to note that 76-nt ssDNA is also a good substrate for ngMutL.

**Figure 8:**
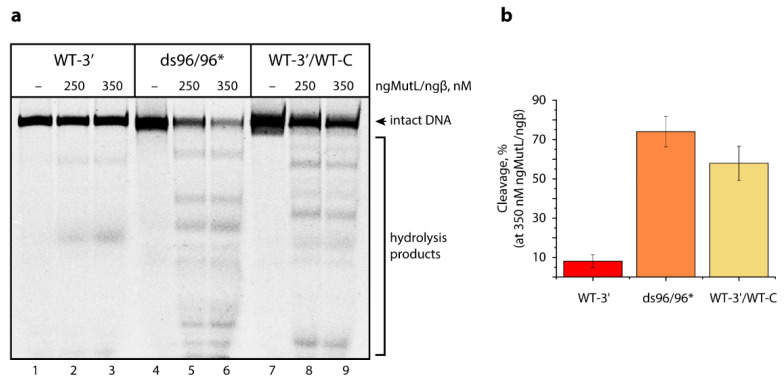
The ngMutL-induced hydrolysis of the WT-3′ *hTERT* G4, ds96/96*, and WT-3′/WT-C (10 nM concentration). The reaction mixtures were incubated in the presence of 0.8 mM ATP, 5 mM MgCl_2_, and 5 mM MnCl_2_ for 90 min at 37 °C, and then electrophoresed in a 12% polyacrylamide gel containing 7 M urea; 0.25 μM ngMutL or 0.35 μM ngMutL (per dimer) was used in the presence of an equimolar amount of ngβ dimer. (**a**) Electropherogram of TAMRA-labeled DNA cleavage products. (**b**) Hydrolysis efficiency in the presence of 0.35 μM proteins.

To understand the role of tandem three-quadruplex structure in the promoter *hTERT* for the efficiency of ngMutL-induced DNA cleavage, we compared the substrate activity of 3′-TAMRA-labeled ds96/96* without G4 structure and the WT-3′ *hTERT* G4 of the same length, as well as the double-stranded version of the WT-3′ *hTERT* G4 formed by hybridization with a fully complementary oligonucleotide: dsWT-3′/WT-C (Supplementary Table S1). Interestingly, the efficiency of the ngMutL-mediated hydrolysis of the WT hTERT G4 is reduced compared to the duplex ds96/96* DNA lacking the G4 structure (Figure 8). The hydrolysis of dsWT-3′/WT-C, as well as ds96/96*, is rather efficient and non-specific, confirming the complete disappearance of the G4 structure after the addition of a fully complementary strand.

**Figure 9:**
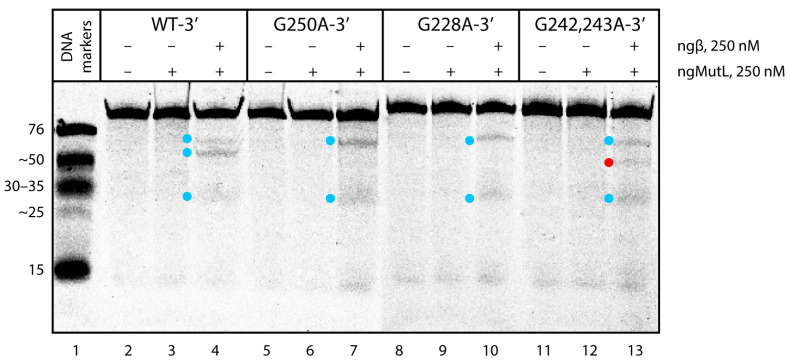
The ngMutL-induced hydrolysis of WT-3′ *hTERT* G4 and its altered forms. The reaction conditions are the same as in the legend in Figure 8; 250 nM ngMutL (per dimer) was used in the presence of an equimolar amount of ngβ dimer. The lengths of DNA markers (in nucleotide residues) are shown on the left; TAMRA-labeled DNA cleavage products are indicated by blue and red dots.

The next task was to understand whether the G>A substitutions in the central G4 unit of the 96-nt *hTERT* G4 structure affect the extent and direction of the DNA hydrolysis induced by ngMutL. As evident from the results, ngMutL cleaves the *hTERT* G4 altered forms with a potency and specificity almost similar to those demonstrated by WT-3′ (Figure 9). The analysis of the electropherogram using the TotalLab CLIQS 1.5 program revealed the presence of two zones of weak cleavage (indicated by blue dots in Figure 9), corresponding to products ranging in size from 30- to 35-nt and a little less than 76-nt. These positions correspond to linker sequences between the first and second, and the second and third G4s, respectively, in a compact conformation of three stacked parallel G4s. This additionally confirms that the G4 structure is not destroyed as a result of point nucleotide substitutions. However, in the case of double substitution (G242,243A), a weak additional band was found corresponding to the oligonucleotide cleavage product of about 45 nt, corresponding to the position of the G243A substitution (indicated by red dot in Figure 9). The data support local G4 destabilization in this substrate, leading to the ngMutL-induced DNA cleavage in the region of the G>A double substitution. The hydrolysis of the double-stranded versions of the *hTERT* containing G>A substitutions, formed by hybridization with fully complementary oligonucleotides, was as efficient as the hydrolysis of the WT-3′/WT-C (Supplementary Figure S9).

It should be noted that an increase in the concentration of ngMutL and ngβ dimers to 0.5 µM resulted in the almost 100% hydrolysis of 10 nM ds- or ssDNAs lacking the G4 structure. However, this factor had no effect on the hydrolysis efficiency of the WT *hTERT* G4 and its altered forms (data not shown).

Thus, it was shown that the multiquadruplex structure of the G-rich strand of the *hTERT* promoter does not prevent the binding of ngMutL to DNA substrates (Table 2), but rather affects the efficiency of the DNA cleavage by this enzyme. Regardless of the G>A substitutions in the central G4 core, the stable G4 scaffold leads to the inhibition of ngMutL nuclease activity, as the cleavage points are located not in G4 itself, but in the regions flanking this noncanonical structure.

## 4. Discussion

Point mutations in the *hTERT* gene promoter occur at a high frequency in multiple cancers, but the reasons for the extremely high mutation frequency in the noncoding regions of the human genome remain unclear. hTERT is the catalytic subunit of telomerase, an enzyme primarily responsible for the immortality of malignant cells [1]. In this work, we studied the G4-forming potential of the 96-nt ssDNA model of the G-rich *hTERT* gene promoter region with 12 consecutive G-tracts, as well as its three analogs containing single or double G>A substitutions corresponding to the three most known driver mutations. These DNA models were then used to investigate whether *hTERT* G4s could influence key properties of the MutS and MutL proteins involved in the initial stages of the MMR process. We suggest that the G4 formation may have a negative impact on mismatch repair, leading to a high mutation rate in the G/C-rich regions of oncogene promoters.

The human genome contains about half a million sites capable of forming G4 structures. Such noncanonical DNA, often localized in the promoters of oncogenes, in translocation hot spots, etc., have received much attention with respect to determining the roles these structures play in the biological regulation of health and disease. In recent years, G4s have been considered as anti-cancer targets for clinical applications [24,53,54].

The folding pattern of the WT hTERT G4 and its analogs with different cancer-associated nucleotide substitutions in the G4 motif was examined by DMS chemical probing. To obtain more reliable data, TAMRA-labeled oligonucleotides with a fluorescent label located either at the 5′ or 3′ end of the molecule were used. The results obtained strongly suggest that the WT *hTERT* promoter sequence does not form two stacked quadruplexes, including one with an unusual DNA hairpin, but rather folds into a compact structure containing three stacked parallel G4 units (Figure 1).

These results are consistent with recently published data [55]. We have shown, for the first time, that such higher-order G4 structures can be successfully examined by chemical probing with DMS. The substituted *hTERT* promoter sequences were found to form only slightly different G4 structures; G>A point substitutions in the G-tracts corresponding to the central quadruplex unit destabilize to some extent, but do not destroy the compact conformation of tandem G4s. However, this is true for isolated ssDNA. In vivo, in the presence of a complementary strand even under conditions favorable for G4 stabilization, the energetically unfavorable G4 folding of the *hTERT* promoter region, carrying single G>A substitutions in the G-tracts, can shift the G4–double helix equilibrium towards duplex formation, and accordingly, change the efficiency and mode of DNA interaction with repair proteins. It should be noted that the effect of G>A substitutions on the folding and stability of the *hTERT* G4 structure was studied earlier using the same approach [56]. However, the authors used an incomplete *hTERT* promoter sequence containing 8 out of 12 G-tracts capable of forming only two G4 units. Nevertheless, they showed, as in our work, an increased sensitivity of the altered G-tract and the adjacent one to DMS treatment.

The CD spectrum of the WT *hTERT* G4 under the conditions of quadruplex formation reveals the characteristic features of a parallel G4 structure with a positive peak at about 265 nm and a negative peak at 240 nm, wherein all the guanosines in the G-tetrads are in *anti*-conformations [57] (Figure 4b). The melting curves of the WT *hTERT* G4 monitored by UV and CD spectroscopy methods demonstrated a single-step cooperative transition, which can be interpreted as a melting superposition of three quadruplexes stabilized by an equal number of G-tetrads participating in continuous *π–π* stacking interactions along three stacked parallel G4 units. The formation of G4 multimers may be a characteristic feature of the promoter quadruplexes, since it is exactly the parallel G4s that tend to “stick” to each other [58,59]. Higher-order G4 structures are currently speculated to be involved in various biological processes, primarily as unique recognition sites for proteins that stabilize or disrupt the G4 stacking interfaces, or as concentration-dependent G4 biological switches.

Given the negative impact of G4s on genome integrity, it is of prime interest to understand their impacts on the repair machinery. Among the identified G4-binding proteins, there are many G4-resolving and repair proteins, such as special helicases and proteins involved in homologous recombination, BER or NER [60,61], as well as DNA mismatch repair [26,27]. Using the characterized *hTERT* G4 DNA models, we examined whether these multiquadruplex structures could influence the key properties of the proteins involved in the initial stages of MMR in two bacterial species, *E. coli* and *N. gonorrhoeae*, having different MMR mechanisms. The main components of the MMR system, MutS and MutL, are highly conserved proteins that are both needed to initiate appropriate DNA repair and DNA damage-signaling responses in a coordinated manner. In *E. coli*, methyl-directed MMR, MutS, and MutL together activate the MutH endonuclease, which nicks the newly synthesized DNA strand at the adjacent 5′-Gm^6^ATC-3′/3′-CTAG-5′ hemimethylated site [62]. In contrast, *N. gonorrhoeae*, similar to many other microorganisms and eukaryotes, including humans, does not contain MutH endonuclease; instead, the MutL homolog exhibits weak endonuclease activity [34]. Therefore, ngMutL can serve as a useful tool for functional studies of eukaryotic MutL properties.

Using bioinformatics tools, we have shown that in addition to common properties, human MutSα and MutLα exhibit a similar 3D structure to the bacterial proteins used in our research (Figure 6). Even though bacterial MutS and MutL are homodimers (instead of heterodimeric eukaryotic ones), the functional subunits behave differently [63]. It has recently been shown that prokaryotic MMR proteins are the evolutionary ancestors of eukaryotic homologs [63]. We believe the results obtained with prokaryotic proteins can be applied to the human repair system.

Using an EMSA assay, it was shown that the difference in ecMutS’s, ecMutL’s, or ngMutL’s binding affinity to the WT *hTERT* G4 and its substituted analogs was negligible, which confirms the formation of closely related G4 structures regardless of the presence of G>A single or double nucleotide substitutions in the central G4 motif and its location (Table 1 and Table 2). Unlike ecMutL and ngMutL, the affinity of ecMutS to G4-containing DNA of similar length increases with the number of G4s. 

The analysis of the ngMutL-mediated cleavage of *hTERT* G4 models revealed that the efficiency of the WT-3′ *hTERT* G4 hydrolysis was markedly reduced compared to the ds96/96 lacking the G4 structure and the double-stranded WT *hTERT* fragment formed by hybridization with fully complementary oligonucleotides, such as WT-3′/WT-C (Figure 8a). The location of the cleavage points corresponds to the linker sequences between the G4 units within the *hTERT* fragment with three stacked parallel G4s. While G228A and G250A substitutions in the central G4 unit of the 96-nt *hTERT* G4 structure have practically no effect on the extent and direction of the DNA hydrolysis induced by ngMutL (Figure 9), in the case of G242,243A, a weak additional band was found corresponding to the position of double G>A substitution, i.e., the site of local G4 destabilization.

Thus, it was shown that the multiquadruplex structure of the *hTERT* promoter does not prevent the effective binding of ngMutL to DNA substrates (Table 2), but rather affects the efficiency of DNA cleavage by this enzyme. Regardless of the G>A substitutions in the central G4 core, the stable G4 scaffold leads to the inhibition of the ngMutL endonuclease activity, the cleavage points being located not in the G4 itself, but in the regions flanking these noncanonical structures, or in the site of the local G4 destabilization. This is in stark contrast to the *E. coli* methyl-directed MMR. Despite the efficient binding of ecMutS and ecMutL to the G4-containing DNA, these MMR proteins do not affect the MutH-induced hydrolysis of a DNA substrate lacking the G/T mismatch but containing the G4 structure stabilized in the duplex surrounding [27].

## 5. Conclusions

The interplay between G4 structures and DNA repair machinery, the activity of which is coupled to genomic stability, has become the subject of intense research [23]. Here, we studied the functional responses of the DNA mismatch repair systems of two bacterial species to the tandem G4 structure formed by the noncoding strand of the *hTERT* promoter and its altered variants bearing point G>A substitutions (at positions 228, 250, 242/243, which are clustered in the middle of G4 motif) associated with cancer-causing driver mutations. Mismatched G/T pairs, which can be effectively repaired by MMR proteins, are formed as intermediates in the transformation of C>T point substitutions (possibly originating from the deamination of 5-methylcytosine or DNA polymerase errors during replication) into C/G>T/A mutations. Our results show that both isolated G4 (in 95G4) and the three-quadruplex tandem (in WT *hTERT* G4 and its variants with point G>A substitutions) significantly suppress the endonuclease activity of ngMutL, a protein of the methyl-independent MMR system inherent in eukaryotes. At the same time, this enzyme, as well as the ecMutS and ecMutL proteins from *E. coli* involved in methyl-directed MMR, exhibit a high binding affinity for all G4-containing DNA substrates whose secondary structure was established by chemical probing and spectroscopic methods. Based on these data, we hypothesize that: (i) the mode of ngMutL action is probably shared with human MutL homologs; (ii) the interaction of MMR proteins with G4 can contribute to the stabilization of the quadruplex structure, the formation of which requires the recruitment of G residue from a mismatched G/T pair located in the *hTERT* promotor region, and as a result, leads to the disruption of the MMR recognition site and the protection of some DNA mismatches from repair. Thus, the G4 structures formed in the oncogene promoters can act as mutagenic factors leading to genomic instability in malignant cells through their interference with MMR process.

## Figures and Tables

**Table 1:** Apparent dissociation constants of ecMutS and ecMutL complexes with ssDNA models containing G4 motifs.

Oligonucleotides	*K*_D_^app^, nM	
	ecMutS	ecMutL
WT-5′	15 ± 6	115 ± 30
G228A-5′	24 ± 6	170 ± 40
G250A-5′	30 ± 7	130 ± 30
G242,243A-5′	27 ± 4	110 ± 20
95G4	38 ± 5	120 ± 20

**Table 2:** Apparent dissociation constants (*K*_D_^app^, nM) for ngMutL complexes with single-stranded 3′-TAMRA-labeled 96-nt WT and altered *hTERT* G4s.

DNAs	*K*_D_^app^, nM
WT-3′	49 ± 3
G228A-3′	44 ± 3
G250A-3′	42 ± 2
G242,243A-3′	51 ± 2
95G4	41 ± 1
ds96/96*	125 ± 5

## Data Availability

Data is contained within the article or supplementary material.

## Supplementary Materials

The following supporting information can be downloaded at: https://www.mdpi.com/article/10.3390/biomedicines10081871/s1, Table S1: Primary structures of DNA models used to study the interactions of G4 and MMR proteins; Figure S1: The conserved residues and motifs in different MutS homologs; Figure S2: Comparison of amino acid sequences of MutL protein homologs; Figure S3: Superposition of N-terminal domains of ecMutL, hMLH1, hPMS2; Figure S4: Direct binding of *hTERT* G4 variants bearing the 5′-TAMRA label with ecMutS; Figure S5: Competitive binding of ds76-G/T-3′ bearing the TAMRA label at the 3′-end of the bottom strand from the preformed complex with ecMutS by unlabeled *hTERT* G4 DNA variants; Table S2: IC_50_ values and calculated *K*_I_^app^ values for ecMutS complexes with *hTERT* G4 variants; Figure S6: Direct binding of *hTERT* G4 variants bearing the 5′-TAMRA label with ecMutL; Figure S7: Direct binding of ngMutL to ssDNA models containing G4 motifs and dsDNA control; Figure S8: Cleavage of 3′-TAMRA-labeled 76-nt ssDNA and 76-bp dsDNAs that either do not contain a mismatch or contain a G/T mismatched base pair, by ngMutL in the presence of an equimolar amount of ngβ; Figure S9: ngMutL-induced hydrolysis of duplex WT-3′/WT-C and its altered forms.
