# Supplementary material for: G-Quadruplex Formed by the Promoter Region of the hTERT Gene: Structure-Driven Effects on DNA Mismatch Repair Functions
Source: Biomedicines. 2022 Aug 3;10(8):1871. doi: 10.3390/biomedicines10081871 (PMC9405553; doi:10.3390/biomedicines10081871)
Supplement: Supplementary file 1 [file biomedicines-10-01871-s001.zip › biomedicines-1803148-supplementary.pdf]

Supplementary

Table S1. Primary structures of DNA models used to study the interactions of G4 and MMR proteins. Designations for DNA duplexes are shown on the left, and for single-stranded oligonucleotides are presented on the right.

DNA duplexes	Sequences	Oligonucleotides
–	5'-GCCGCGGAAAGGAA<u>GGGGAGGGGCTGGGAGGGCCCGGAGGGGGCTGGGCCGGGGACCCGGGAGGGGTCGGGACGGGGCGGGG</u>TCCGCGCGGAGGAG-3'	WT
WT-5'/WT-C	TAMRA-5'-GCCGCGGAAAGGAA<u>GGGGAGGGGCTGGGAGGGCCCGGAGGGGGCTGGGCCGGGGACCCGGGAGGGGTCGGGACGGGGCGGGG</u>TCCGCGCGGAGGAG-3'<br>3'-CGGCGCCTTTCCTTCCCCTCCCCGACCCTCCCGGGCCTCCCCCGACCCGGCCCCTGGGCCCTCCCCAGCCCTGCCCCGCCCCAGGCGCGCCTCCTC-5'	WT-5'<br>WT-C
WT-3'/WT-C	5'-GCCGCGGAAAGGAA<u>GGGGAGGGGCTGGGAGGGCCCGGAGGGGGCTGGGCCGGGGACCCGGGAGGGGTCGGGACGGGGCGGGG</u>TCCGCGCGGAGGAG-3'-TAMRA<br>3'-CGGCGCCTTTCCTTCCCCTCCCCGACCCTCCCGGGCCTCCCCCGACCCGGCCCCTGGGCCCTCCCCAGCCCTGCCCCGCCCCAGGCGCGCCTCCTC-5'	WT-3'<br>WT-C
G228A-5'/C228T	TAMRA-5'-GCCGCGGAAAGGAA<u>GGGGAGGGGCTGGGAGGGCCCGGA**A**GGGGCTGGGCCGGGGACCCGGGAGGGGTCGGGACGGGGCGGGG</u>TCCGCGCGGAGGAG-3'<br>3'-CGGCGCCTTTCCTTCCCCTCCCCGACCCTCCCGGGCCTTCCCCGACCCGGCCCCTGGGCCCTCCCCAGCCCTGCCCCGCCCCAGGCGCGCCTCCTC-5'	G228A-5'<br>C228T
G228A-3'/C228T	5'-GCCGCGGAAAGGAA<u>GGGGAGGGGCTGGGAGGGCCCGGA**A**GGGGCTGGGCCGGGGACCCGGGAGGGGTCGGGACGGGGCGGGG</u>TCCGCGCGGAGGAG-3'-TAMRA<br>3'-CGGCGCCTTTCCTTCCCCTCCCCGACCCTCCCGGGCCTTCCCCGACCCGGCCCCTGGGCCCTCCCCAGCCCTGCCCCGCCCCAGGCGCGCCTCCTC-5'	G228A-3'<br>C228T
G250A-5'/C250T	TAMRA-5'-GCCGCGGAAAGGAA<u>GGGGAGGGGCTGGGAGGGCCCGGAGGGGGCTGGGCCGGGGACCCGG**A**AGGGGTCGGGACGGGGCGGGG</u>TCCGCGCGGAGGAG-3'<br>3'-CGGCGCCTTTCCTTCCCCTCCCCGACCCTCCCGGGCCTCCCCCGACCCGGCCCCTGGGCCTTCCCCAGCCCTGCCCCGCCCCAGGCGCGCCTCCTC-5'	G250A-5'<br>C250T
G250A-3'/C250T	5'-GCCGCGGAAAGGAA<u>GGGGAGGGGCTGGGAGGGCCCGGAGGGGGCTGGGCCGGGGACCCGG**A**AGGGGTCGGGACGGGGCGGGG</u>TCCGCGCGGAGGAG-3'-TAMRA<br>3'-CGGCGCCTTTCCTTCCCCTCCCCGACCCTCCCGGGCCTCCCCCGACCCGGCCCCTGGGCCTTCCCCAGCCCTGCCCCGCCCCAGGCGCGCCTCCTC-5'	G250A-3'<br>C250T
G242,243A-5'/C242,243T	TAMRA-5'-GCCGCGGAAAGGAA<u>GGGGAGGGGCTGGGAGGGCCCGGAGGGGGCTGGGCCGG**AA**ACCCGGGAGGGGTCGGGACGGGGCGGGG</u>TCCGCGCGGAGGAG-3'<br>3'-CGGCGCCTTTCCTTCCCCTCCCCGACCCTCCCGGGCCTCCCCCGACCCGGCCTTTGGGCCCTCCCCAGCCCTGCCCCGCCCCAGGCGCGCCTCCTC-5'	G242,243A-5'<br>C242,243T
G242,243A-3'/C242,243T	5'-GCCGCGGAAAGGAA<u>GGGGAGGGGCTGGGAGGGCCCGGAGGGGGCTGGGCCGG**AA**ACCCGGGAGGGGTCGGGACGGGGCGGGG</u>TCCGCGCGGAGGAG-3'-TAMRA<br>3'-CGGCGCCTTTCCTTCCCCTCCCCGACCCTCCCGGGCCTCCCCCGACCCGGCCTTTGGGCCCTCCCCAGCCCTGCCCCGCCCCAGGCGCGCCTCCTC-5'	G242,243A-3'<br>C242,243T
ds76-A/T-3'	5'–ATAGGACGCTGACACTGGTGCTTGGCAGCTGAGCCATATGCTCGAGTAACGCTCATAGGATCCAAGCGCGAAAGGA–3'–TAMRA<br>3'–TATCCTGCGACTGTGACCACGAACCGTCGACTCGGTATACGAGCTCATTGCGAGTATCCTAGGTTCGCGCTTTCCT–5'	ss76<br>76A
ds76-G/T-3'	5'–ATAGGACGCTGACACTGGTGC**T**TGGCAGCTGAGCCATATGCTCGAGTAACGCTCATAGGATCCAAGCGCGAAAGGA–3'–TAMRA<br>3'–TATCCTGCGACTGTGACCACG**G**ACCGTCGACTCGGTATACGAGCTCATTGCGAGTATCCTAGGTTCGCGCTTTCCT–5'	ss76<br>76G
ds96/96*	5'-ACCTGGATGCCTATAGGGCGAATTGGGTACCGCTGAATTGCACTGGACTTGATCCTCGATGATCCTAAGCTAAGCTTCAGCTCCAGCCTAAGCCTG-3'-TAMRA<br>3'-TGGACCTACGGATATCCCGCTTAACCCATGGCGACTTAACGTGACCTGAACTAGGAGCTACTAGGATTCGATTCGAAGTCGAGGTCGGATTCGGAC-5'	96<br>96*
-	5'–ATAGGACGCTGACACTGGTGCTTGGCAGCTGAGCCATATTT<u>GGGTGGGTGGGTGGG</u>TTGCTCGAGTAACGCTCATAGGATCCAAGCGCGAAAGGA-3'–TAMRA	95G4

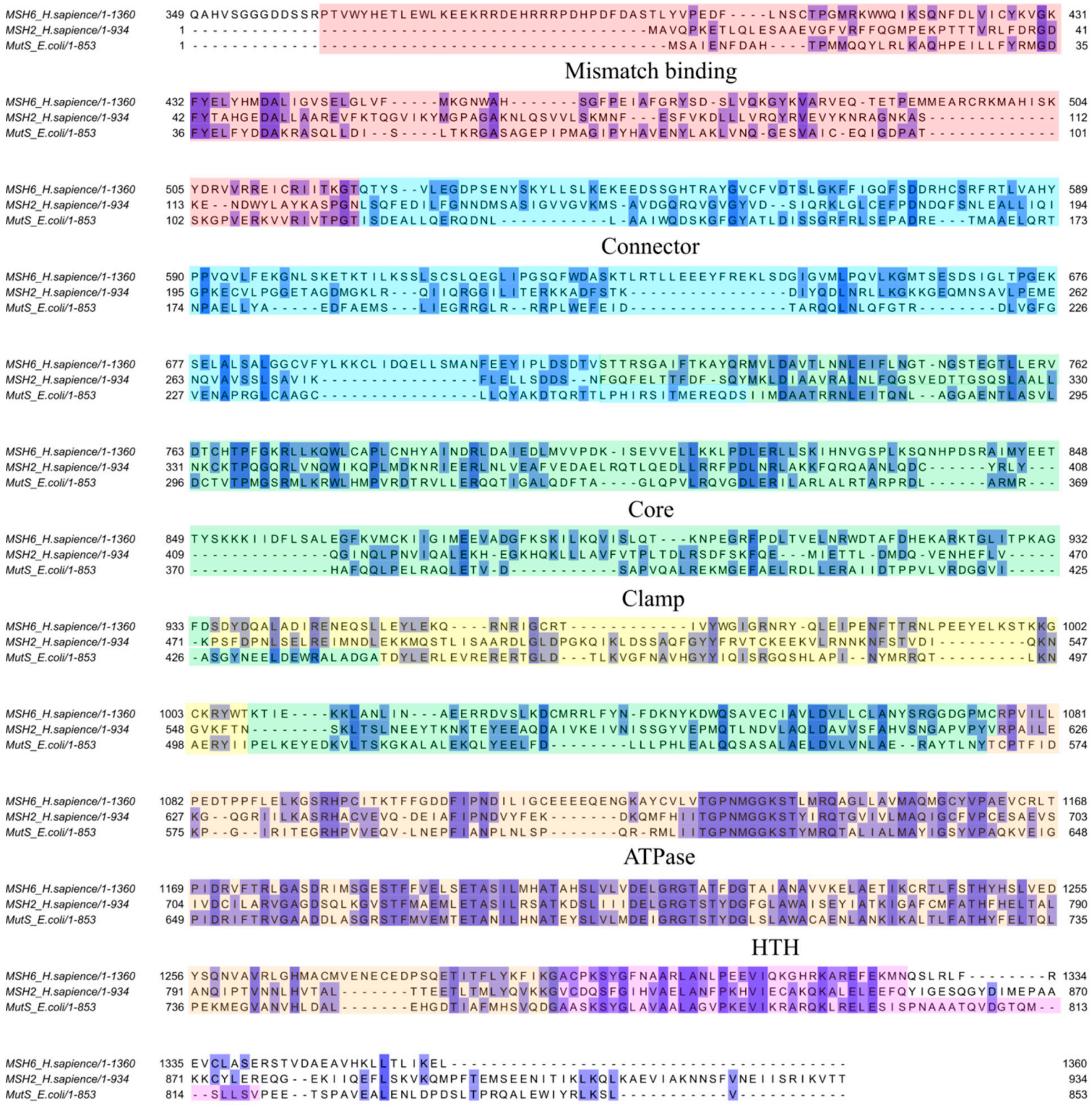

Figure S1. Conserved residues and motifs in different MutS homologs: ecMutS (UniProtKB - P23909), hMSH6 (UniProtKB - P52701), and hMSH2 (UniProtKB - P43246). Alignment was done using the ClustalO algorithm with default settings. Identical residues are marked in blue. The domains of MutS homologs are distinguished according to Sachadyn [36] and marked with different background colors.

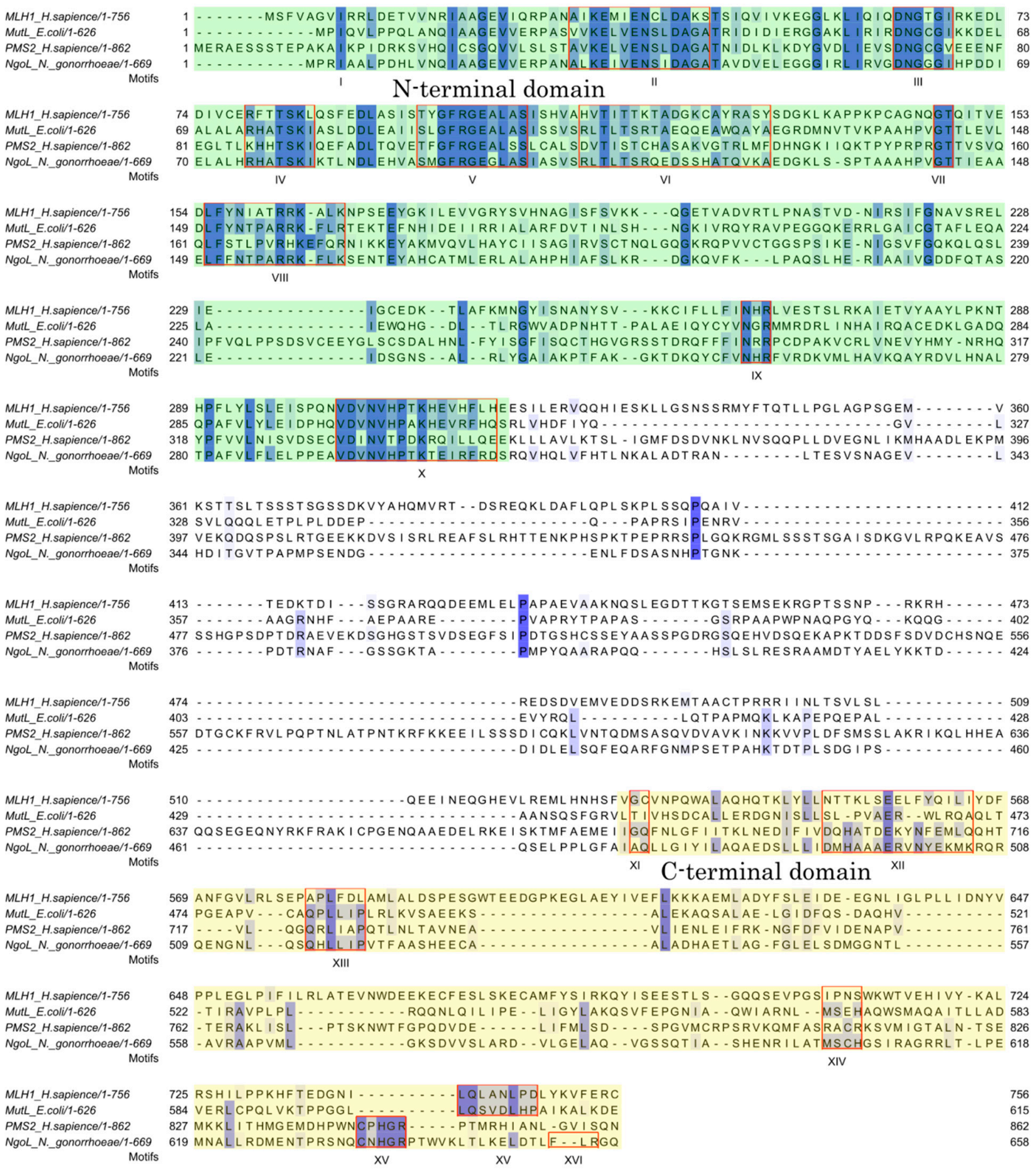

Figure S2. Comparison of amino acid sequences of MutL homologs: ecMutL K12 (UniProtKB - P23367), ngMutL FA 1090 (UniProtKB - Q5F8M6), hMLH1 (UniProtKB - P40692) and hPMS2 (UniProtKB - P54278). Identical residues are marked in blue. N- and C-domains of MutL homologs are distinguished and marked with light green and yellow background, respectively. Linker regions (not signed) are located between the N- and C-domains. Conserved MutL motifs of are indicated in red boxes [37].

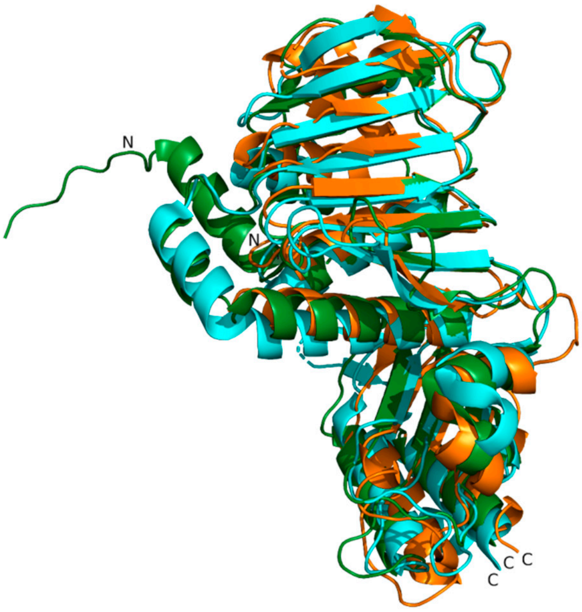

Figure S3. Overlay of N-terminal domains of ecMutL (PDB ID 1BKN, orange), hMLH1 (PDB ID 3NA3, green), hPMS2 (PDB ID 1H7U, sky blue).

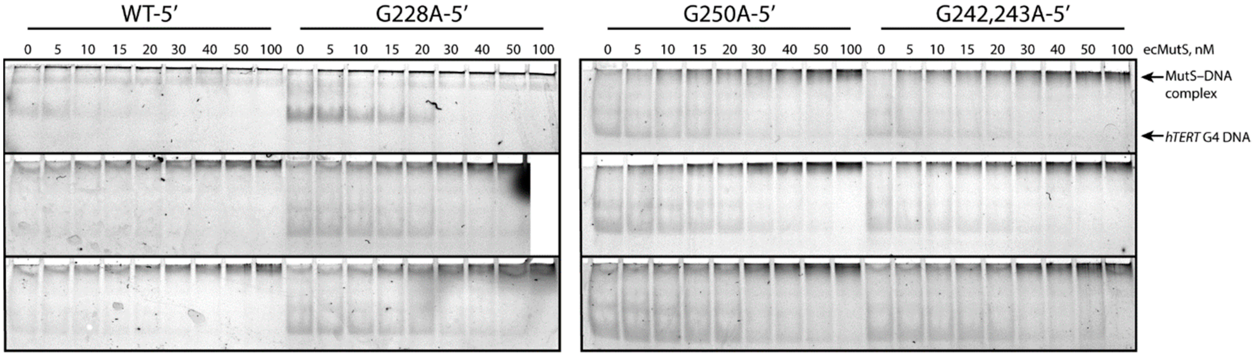

Figure S4. Direct binding of *hTERT* G4 variants 5'-labeled with TAMRA (5 nM) to ecMutS (0–100 nM per protein dimer). The efficiency of complex formation was assessed using EMSA assay in non-denaturing 6% polyacrylamide gel. Three repeats for each *hTERT* G4 variant are shown in three rows.

Competitive binding of ecMutS to fluorescently labeled 76-bp DNA containing G/T mismatch in the presence of *hTERT* G4s.

We performed competition experiments using a G/T mismatch-containing 76-bp DNA duplex (ds76-G/T-3') (Table S1) bearing the TAMRA fluorophore at the 3'-end of the bottom strand as the preferred substrate for ecMutS; the K_d^{app} of the ecMutS•ds76-G/T-3' complex being 35±3 nM. This complex was then co-incubated with increasing concentrations of either unlabeled WT *hTERT* G4 or its G>A substituted analogs. The amount of displaced ds76-G/T-3' was assessed using EMSA assay. The IC_{50} values found for each competitor were used to calculate the dissociation constants (K_I^{app}) of their complexes with ecMutS using the equation: $K_I^{app} = \frac{IC_{50}}{1+[L]/K_D^{app}}$, where [L] is a concentration of ds76-G/T-3' and K_D^{app} is the apparent dissociation constant of the ecMutS• ds76-G/T-3' complex.

In this setup, the affinities of ecMutS to all investigated *hTERT* G4s were close, and K_I^{app} lied in the range of 25–35 nM (Table S2), which in most cases coincided with the error range compared to direct binding. The IC_{50} values corresponded to the *hTERT* G4 concentrations at which 50% of the competitor DNA ligand interacting with the ecMutS were averaged over at least three independent experiments. The errors are presented as 95% confidence intervals.

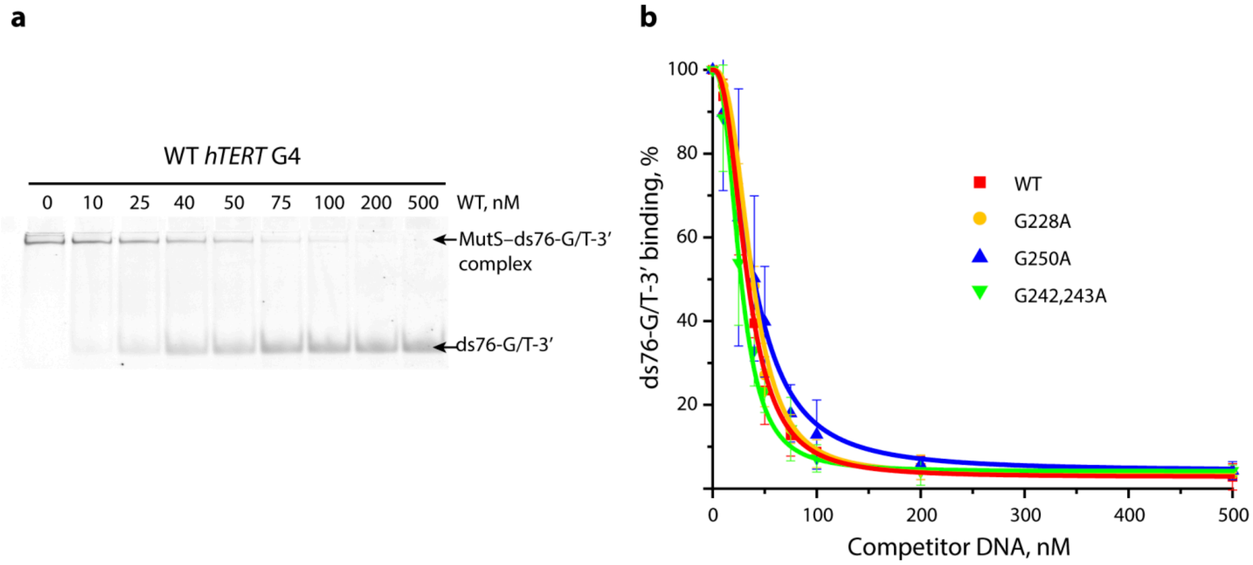

Figure S5. Competitive displacement of ds76-G/T-3' bearing the TAMRA label at the 3'-end of the bottom strand from a preformed complex with ecMutS (65 nM per protein dimer) by unlabeled *hTERT* G4s (0–500 nM). (**a**) The amount of displaced ds76-G/T-3' by increasing WT *hTERT* G4 concentration was assessed using EMSA assay in 6% polyacrylamide gel unred nondenaturing conditions. (**b**) Curves of competitive displacement of ds76-G/T-3' from its complex with ecMutS (5 nM ds76-G/T-3', 65 nM protein, 1 mM ADP) by WT and altered forms of *hTERT* G4 (0–500 nM).

Table S2. IC_{50} values and calculated K_I^{app} values for ecMutS complexes with *hTERT* G4 variants.

**Oligonucleotides**	**Competition binding characteristics**	
	**IC_{50}, nM**	**K_I^{app}, nM**
WT	32±3	28±5
G228A	35±4	31±6
G250A	40±15	35±15
G242,243A	28±7	25±8

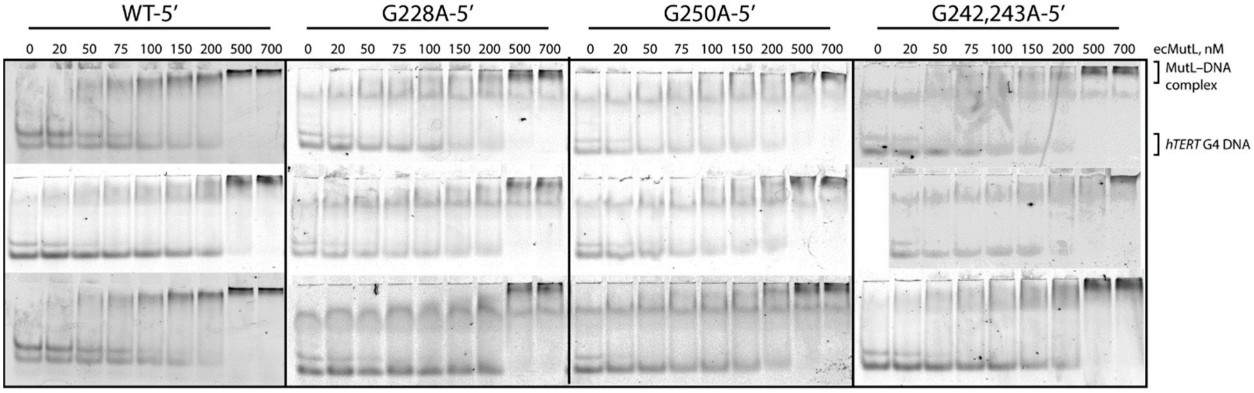

Figure S6. Direct binding of *hTERT* G4 variants bearing the 5'-TAMRA label (20 nM) to ecMutL (0–700 nM per protein dimer). The efficiency of complex formation was evaluated by EMSA assay in a non-denaturing 4% polyacrylamide gel. Three repeats for each *hTERT* G4 variant are shown in three rows.

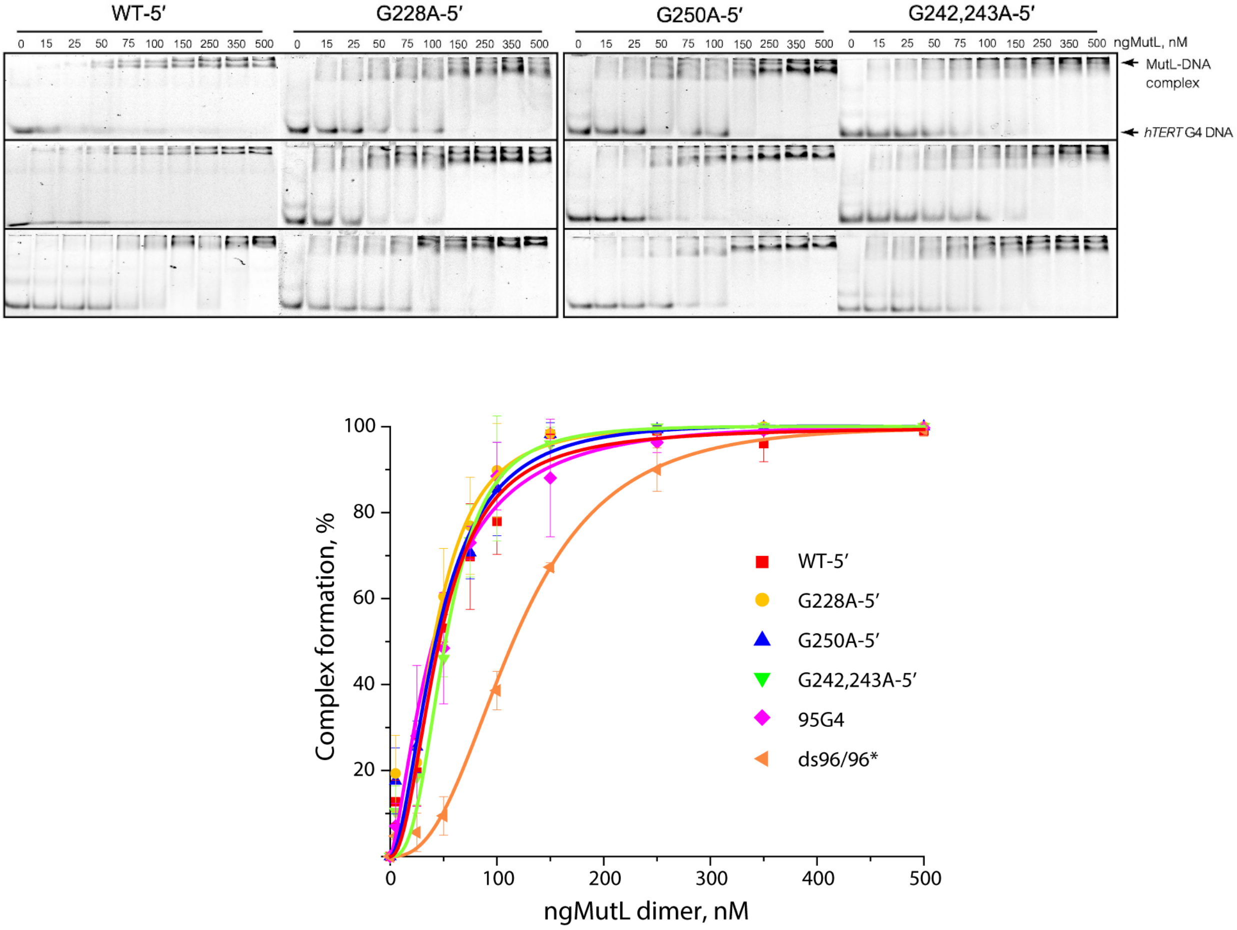

Figure S7. Direct binding of ngMutL to ssDNA models containing G4 motifs and control dsDNA. Electrophoregrams of ngMutL binding to *hTERT* G4 variants are shown above. Three repeats for each *hTERT* G4 variant are shown in three rows. Below, the yield of nucleic acid–protein complexes calculated from EMSA data is plotted against the total ngMutL concentration (0–1000 nM per ngMutL dimer at 20 nM DNA).

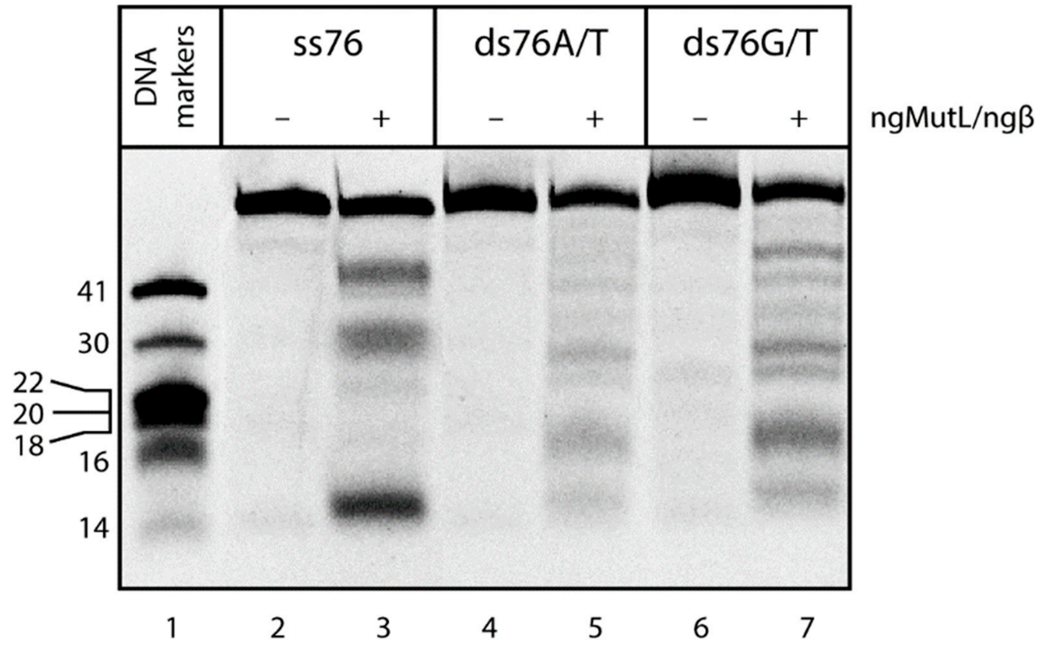

Figure S8. Cleavage of 10 nM 3'-TAMRA-labeled 76-nt ssDNA (lanes 2 and 3) and 76-bp dsDNAs, which either do not contain (ds76-A/T-3') (lanes 4, 5) or contain a G/T mismatched base pair (ds76-G/T-3') (lanes 6, 7), by 250 nM ngMutL (per dimer) in the presence of an equimolar amount of ngβ. The reaction mixtures were also incubated in the presence of 0.8 mM ATP, 5 mM $MgCl_2$ and 5 mM $MnCl_2$ for 90 min at 37 °C, and then analyzed in a 12% polyacrylamide gel containing 7 M urea. The length of DNA markers (in nucleotide residues) are shown on the left.

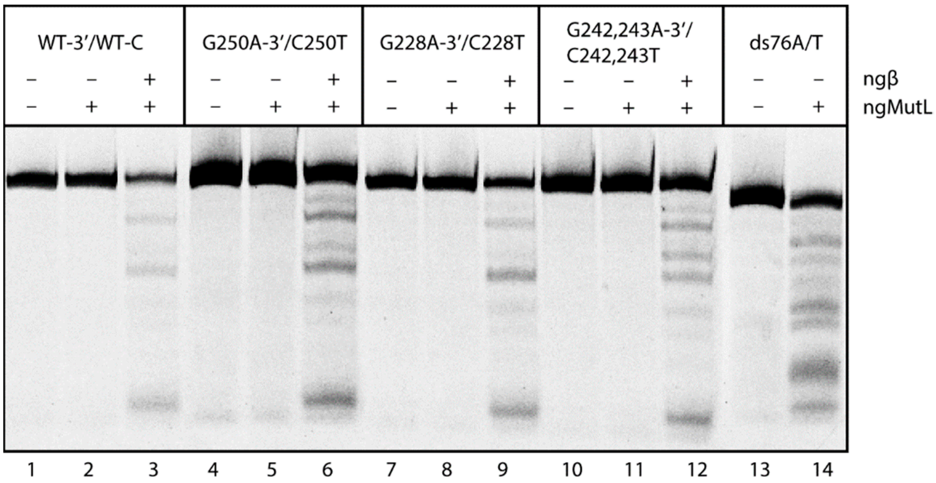

Figure S9. ngMutL-induced hydrolysis of duplex WT-3'/WT-C and its altered forms. The reaction conditions are the same as in the legend to Figure 8; 250 nM ngMutL (per dimer) was used in the presence of an equimolar amount of ngβ. ds76A/T DNA was used in the control experiment.
